# Acupoint catgut embedding for diabetic gastroparesis: A protocol of systematic review

**DOI:** 10.1097/MD.0000000000017718

**Published:** 2019-10-25

**Authors:** Tingwei Xia, Yue Yang, Weihong Li, Zhaohui Tang, Zongrun Li, Yongsong Guo

**Affiliations:** a Basic Medical College, Chengdu University of TCM; b Department of TCM, Qingyang District People's Hospital, Chengdu, Sichuan Province, China.

**Keywords:** acupoint catgut embedding, diabetic gastroparesis, meta-analysis, systematic review

## Abstract

**Background:**

Diabetic gastroparesis (DG) is a common complication to diabetes mellitus (DM). A lot of clinical researches have focused on acupoint catgut embedding for diabetic gastroparesis. However, there is no systematic review and meta-analysis has been conducted. We aim to systematically review the effect of acupoint catgut embedding on diabetic gastroparesis.

**Methods:**

The databases including PubMed, Cochrane Central Register of Controlled Trials (CENTRAL), Web of Science, Ovid LWW, EMBASE, CNKI, Wanfang Database will be searched. Studies published from the time when the database establishment to July 2019 will be retrieved. Randomized controlled clinical trials and quasi-randomized controlled trials on acupoint catgut embedding for diabetic gastroparesis will be included. The primary outcomes are gastroparesis Cardinal Symptom Index (GCSI) or a similar scale to score dyspeptic symptoms. RevMan V.5.3 software will be used to perform the analyses.

**Results:**

This study will provide a high-quality synthesis of the effectiveness and safety of acupoint catgut embedding for diabetic gastroparesis.

**Conclusion:**

This systematic review will provide a high-quality evidence to judge whether the acupoint catgut embedding is beneficial to treat diabetic gastroparesis.

**PROSPERO registration number:**

CRD42019140951

## 1 Introduction

Diabetic gastroparesis (DG) is a common complication to DM often associated with autonomic neuropathy and poorly controlled hyperglycemia.^[1]^ Due to the delayed gastric emptying, typical symptoms of DG include nausea, early satiety, vomiting of undigested food, and prolonged fullness.^[2–5]^ According to one study, the prevalence of DG was estimated to be 5% among patients with type 1 diabetes mellitus, 1% among patients with type 2 diabetes mellitus, and 0.2% in non-diabetic controls.^[6–9]^ It was reported there were approximately 451 million people (age 18–99 years) with DM in 2017. It is estimated by the IDF there will be 629 million people with diabetes after 30 years.^[10]^ As the prevalence of DM increases, the number of DG will continue to rise. The heavy economic burden to individuals and society has been caused by diabetic gastroparesis. Diabetes mellitus is the most commonly recognized systemic disease associated with gastroparesis. As the US National Inpatient Sample database reported, the aggregate charges for patients with gastroparesis were $568 million in 2013.^[11]^

Since the delayed gastric emptying is considered as a potential contributor to this functional dyspepsia, conventional medications for diabetic gastroparesis are gastric stimulants such as domperidone, cisapride, and mosapride.^[12–14]^ Acupoint catgut embedding, characterized by strong stimulation, easy operation and durable, and the long interval between each treatment, is developed from TCM acupuncture.^[15]^ It refers to use a certain section of absorbable catgut suture implanted in acupoint. Several systematic reviews or meta-analyses have shown that acupoint catgut embedding is beneficial to obesity, diabetes, psoriasis, and so on.^[16–18]^ In recent years, more and more studies focused on the efficiency of acupoint catgut embedding in the treatment of gastroparesis. However, there is no systematic review of the therapeutic effect of acupoint catgut embedding on diabetic gastroparesis. Therefore, this systematic review and meta-analysis is conducted to evaluate the effectiveness of acupoint catgut embedding for diabetic gastroparesis.

## 2 Methods

### 2.1 Inclusion criteria

#### 2.1.1 Types of participants

Participants diagnosed as diabetes with dyspeptic symptoms will be included. Those with gastric outlet obstruction or ulceration by upper endoscopy, ultrasound or barium X-ray, will be excluded. All participants will regardless of age, gender and ethnicity.

#### 2.1.2 Types of interventions and comparisons

Interventions in the observation group included simple acupoint catgut embedding and acupoint catgut embedding combined with other therapies. The control group was treated with sham acupuncture or gastroprokinetic agents.

#### 2.1.3 Types of outcomes

The primary outcome measurement was gastroparesis Cardinal Symptom Index (GCSI) or a similar scale to score dyspeptic symptoms. The secondary outcome measure was gastric emptying detected by scintigraphy or radio-opaque markers. The adverse events as safety outcomes will be reported.

#### 2.1.4 Types of studies

Randomized controlled clinical trials and quasi-randomized controlled trials will be included.

### 2.2 Data sources and selection strategy

The literature search was performed using the following databases: PubMed, Cochrane Central Register of Controlled Trials (CENTRAL), Web of Science, Ovid LWW, EMBASE, CNKI, Wanfang Database. No language restrictions are imposed. Studies published from the time when the database establishment to July 2019 will be retrieved. An illustrative PubMed search strategy is as follows: diabetic gastroparesis; gastrointestinal changes; gastrointestinal disease; acupoint catgut embedding; catgut embedding; catgut implantation.

### 2.3 Data selection

**Figure 1:**
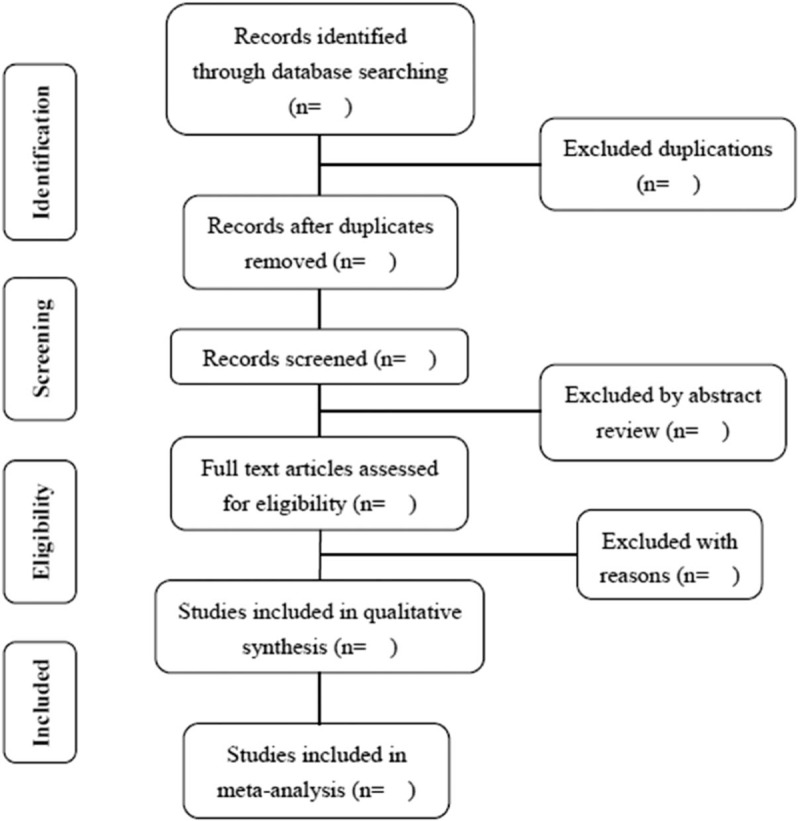
Flow diagram of study selection.

First, appropriate studies will be searched and screened by two independent investigators after reading the titles, abstracts. Next, the same investigators will evaluate the full texts, blinded to each other's review. Disagreements will be resolved by discussion between the authors. The process of study selection will be performed using the methods according to the PRISMA guidelines, presenting in the flow diagram (Fig. 1).^[19,20]^

### 2.4 Data extraction

Two independent reviewers will extract data from the included studies. The data extraction is conducted by using pre-piloted, standardized forms. The data extraction form contains basic information; methodological characteristics; participants’ demographic details; interventions details; outcomes; follow-up; and others. Finally, any disagreements will be resolved by consensus.

### 2.5 Methodological quality of assessment

Two investigators will independently assess the risk of bias according to the “bias risk” assessment tool of the Cochrane Handbook (V.5.1.0).^[21]^ Seven domains should be evaluated, such as random sequence generation, allocation concealment, blinding of participants and investigators, the blindness of outcome assessments, incomplete outcome data, selective outcome reporting, and other biases. Based on the assessment, the quality of studies will be classified into low, unclear, or high bias.

### 2.6 Statistical analysis

The data synthesis will be performed by using the RevMan V.5.3. Dichotomous data will be presented as relative risk (RR). We will express continuous variables as the st. mean ± standard deviation and then calculate the standardized mean difference (SMD) and obtain the 2-sided *P* value and 95% confidence interval (CI). We will use the complete case data as the analysis data.

### 2.7 Assessment of heterogeneity

The heterogeneity of data will be tested by calculating the I^2^ value. If I^2^ < 50%, the fixed-effect model is suitable to be employed to obtain synthesis results for studies with low heterogeneity. If I^2^ ≥ 50%, the random-effects model is adopted to assess the effect size for studies with significant heterogeneity.

We will perform subgroup analysis, as the meta-analysis of the primary outcomes shows significant heterogeneity. Subgroup analysis will be based on different types of acupoint catgut embedding therapy, treatment duration, and curative effects.

### 2.8 Publication bias

If studies included are more than 10 trials, a funnel plot will be used to evaluate potential publication bias. The symmetrical funnel plots will indicate low risk, and asymmetrical funnel plots will indicate a high risk of publication bias.

## 3 Discussion

Syndrome differentiation is one of the traditional characteristics of traditional Chinese medicine, including the identification of lesion site, lesion properties, and degree of seriousness of an illness, emphasizing on the extent and progress of the disease. TCM and Tai Chi, which originated from China, are considered a modality of complementary medicine in Western countries.^[22]^ Acupoint catgut embedding is a part of the routine in the treatment of diabetic gastroparesis in China and is attracting increasing attention.^[23,24]^ And the unusual complications related to acupoint catgut embedding also existed.^[25]^ Previous systematic reviews or meta-analyses have shown that acupoint catgut embedding is beneficial to type 2 diabetes, obesity, psoriasis, and so on.

However, no systematic and comprehensive meta-analysis on the therapeutic effect of acupoint catgut embedding on diabetic gastroparesis has been found. Therefore, we intend to conduct a systematic review of acupoint catgut embedding for diabetic gastroparesis to provide high-quality evidence of effectiveness and safety of acupoint catgut embedding for diabetic gastroparesis, and provide a reference for health policymakers and scientific researchers.

## Author contributions

**Conceptualization:** Tingwei Xia, Yue Yang, Weihong Li.

**Data curation:** Zongrun Li.

**Funding acquisition:** Weihong Li.

**Investigation:** Yue Yang.

**Methodology:** Tingwei Xia, Yue Yang.

**Project administration:** Tingwei Xia, Weihong Li.

**Software:** Yongsong Guo.

**Supervision:** Zhaohui Tang.

**Validation:** Weihong Li.

**Writing – original draft:** Tingwei Xia, Yue Yang.
